# High-resolution computed tomography features associated with differentiation of tuberculosis among elderly patients with community-acquired pneumonia: a multi-institutional propensity-score matched study

**DOI:** 10.1038/s41598-022-11625-7

**Published:** 2022-05-06

**Authors:** Kosaku Komiya, Mari Yamasue, Akihiko Goto, Yuta Nakamura, Kazufumi Hiramatsu, Jun-ichi Kadota, Seiya Kato

**Affiliations:** 1 Department of Respiratory Medicine and Infectious Diseases, Oita University Faculty of Medicine, 1-1 Idaigaoka, Hasama-machi, Yufu, Oita 879-5593 Japan; 2 Department of Respiratory Medicine, Tenshindo Hetsugi Hospital, 5956 Nihongi, Nakahetsugi, Oita 879-7761 Japan; 3 Internal Medicine, National Hospital Organization Nishi-Beppu Hospital, 4548 Tsurumi, Beppu, Oita 874-0840 Japan; 4 Department of Medical Safety Management, Oita University Faculty of Medicine, 1-1 Idaigaoka, Hasama-machi, Yufu, Oita 879-5593 Japan; 5 Nagasaki Harbor Medical Center, 6-39 Shinchi-machi, Nagasaki, 850-8555 Japan; 6 Research Institute of Tuberculosis, Japan Anti-Tuberculosis Association, 3-1-24 Matsuyama, Kiyose, Tokyo 204-8533 Japan

**Keywords:** Diseases, Medical research, Signs and symptoms

## Abstract

While high-resolution computed tomography (HRCT) is increasingly performed, its role in diagnosing pulmonary tuberculosis (TB) among elderly patients with community-acquired pneumonia (CAP) has not been fully elucidated. This study aimed to determine HRCT features that can differentiate pulmonary TB from non-TB CAP in elderly patients. This study included consecutive elderly patients (age > 65 years) admitted to two teaching hospitals for pulmonary TB or non-TB pneumonia who met the CAP criteria of the American Thoracic Society/Infectious Diseases Society of America guidelines. After propensity score matching for clinical background between patients with pulmonary TB and those with non-TB CAP, their HRCT features were compared. This study included 151 patients with pulmonary TB and 238 patients with non-TB CAP. The presence of centrilobular nodules, air bronchograms, and cavities and the absence of ground-glass opacities and bronchial wall thickening were significantly associated with pulmonary TB. The negative predictive values of centrilobular nodules, air bronchograms, and cavities for pulmonary TB were moderate (70.6%, 67.9%, and 63.0%, respectively), whereas the positive predictive value of cavities was high (96.6%). In elderly patients, although some HRCT features could differentiate pulmonary TB from non-TB CAP, no useful findings could rule out pulmonary TB with certainty.

## Introduction

While the prevalence and incidence of tuberculosis (TB) have been declining worldwide, the incidence of newly diagnosed TB from endogenous reactivation after a past TB infection remains high in areas where the elderly population is increasing^1^. Elderly people are prone to both pulmonary TB and non-TB community-acquired pneumonia (CAP)^2,3^. Since the clinical presentation and physical examination are similar and nonspecific among elderly patients with both diseases^2,4^, differentiating pulmonary TB from non-TB CAP is challenging.

Chest X-ray (CXR) images are commonly used in suspecting pulmonary TB, but their sensitivity and specificity are not sufficiently high. Pino et al. reported that using a CXR scoring system resulted in a sensitivity of 85.5% and a specificity of 63.9% in diagnosing pulmonary TB^5^. For elderly patients, chest radiological images may reveal atypical features. In fact, it was demonstrated that elderly patients with TB with bedridden status were likely to show non-cavity and non-upper predominant lung distributions^6^.

High-resolution computed tomography (HRCT) is increasingly performed to evaluate lung involvement in detail. Nevertheless, its usefulness in discriminating these diseases has not been fully studied. Yeh et al. reported an HRCT-based scoring system to differentiate smear-positive pulmonary TB from other CAP, including smear-negative pulmonary TB, in the elderly population^7^. They concluded that HRCT could assist in the early diagnosis of most infectious active pulmonary TB using a scoring system. However, the patients’ backgrounds, clinical presentation, and laboratory data may differ between patients with pulmonary TB and those with non-TB CAP. While these clinical differences might help in diagnosing pulmonary TB or non-TB CAP in clinical practice, other validated diagnostic clues are still required especially for cases posing a diagnostic challenge. To resolve these issues, different patient backgrounds and clinical information require statistical adjustments to reduce selection bias when comparing the radiological features of pulmonary TB and non-TB CAP.

From a public health perspective, it is also important to go beyond the differentiation of these diseases. Indeed, atypical pulmonary TB cases mimicking non-TB CAP are increasingly reported^8,9^. The features for ruling out pulmonary TB among patients with CAP should be elucidated to minimize misdiagnosing cases of pulmonary TB as non-TB CAP. A radiological feature-based scoring system may seem complicated when a rapid evaluation is needed in clinical practice. If there are no radiological features with a high negative predictive value (NPV), especially in medium or high TB burden areas, an acid-fast bacilli (AFB) smear test would be required for all elderly patients presenting with CAP.

Chest HRCT is routinely done to evaluate lung involvements in detail for patients with pulmonary TB and non-TB CAP in our institutions; this is fully covered by the universal health insurance in Japan. Therefore, this study aimed to determine HRCT features that can differentiate pulmonary TB in elderly patients with CAP after matching the patient backgrounds in both diseases and to evaluate the power of HRCT features in ruling out pulmonary TB.

## Patients and methods

### Patients and study design

This multi-institutional, retrospective, observational study was performed at the National Hospital Organization Nishi-Beppu Hospital (for patients with smear-positive pulmonary TB) and Tenshindo Hetsugi Hospital (for elderly patients with CAP) in Oita Prefecture, Japan. We included consecutive elderly patients (age > 65 years) admitted to these hospitals between January 2013 and December 2017 for bacteriologically confirmed pulmonary TB or non-TB CAP who underwent chest CT within two weeks before or after admission. CAP was defined as pneumonia based on the criteria of the American Thoracic Society/Infectious Diseases Society of America guidelines^10^. It was diagnosed based on clinical signs and symptoms, including cough, fever, and infiltrates revealed by chest radiography or chest CT. To focus on cases in which differentiating pulmonary TB from non-TB CAP is challenging based solely on clinical presentations, patients with non-TB CAP and those with TB who met the CAP criteria were included in this study. Their clinical characteristics and chest CT features were compared.

This study was performed in accordance with the Declaration of Helsinki, and the study protocol was approved by the institutional ethics committees (approval number, 3-3; approval date, May 12, 2021, at Nishi-Beppu Hospital; approval number, 2039; approval date, April 12, 2021, at Tenshindo Hetsugi Hospital; and approval number, 2045; approval date, March 22, 2021, at Oita University Hospital). An informed consent was waived by the institutional ethics committee of Oita University Hospital because this was a retrospective study. In addition, information about this study was posted in the hospital, offering patients a chance to opt-out. Some patients included in this study already participated in previous studies^6,11–17^ that have different objectives.

### Data collection

Patient data, including their gender, age, body mass index, daily physical activity levels, underlying diseases, laboratory tests, and the presence of respiratory failure, were obtained from clinical records. The collection of patient information and examination of pulmonary performance were routinely done when a patient diagnosed with pulmonary TB or non-TB CAP was admitted to our hospitals. Their daily physical activities were evaluated on admission using a performance status scale^18^. The definition of respiratory failure was SpO_2_ < 90% without oxygen supplementation on admission.

### Evaluation of plain CXR findings

To compare the predictive values of HRCT features, plain CXR findings were evaluated independently by two respiratory medicine specialists with 14 and 17 years of experience (M.Y. and K.K.) who were blinded to laboratory data, clinical features, and patient diagnosis. The features included ground-glass opacities (GGOs), airspace consolidation, nodules, and cavities. The investigators ascertained the distribution of GGOs and/or airspace consolidation in three areas in both lungs.

Chest radiographs were obtained using a digital Hitachi DHF-155HII unit (Hitachi, Tokyo, Japan) with 120 kV tube voltage and 1.8- or 2-m image receptor distance or a digital Shimadzu UD 150L-40E (Shimadzu, Kyoto, Japan) with 120 kV tube voltage and a 20-m image receptor distance. Posterior-anterior views were routinely obtained. When a posterior-anterior view was not available, an anterior–posterior view was substituted.

### Evaluation of chest HRCT findings

A 16-detector row CT scanner (Activion, Toshiba Medical Systems, Tokyo, Japan) and 320-detector row CT scanner (AquilionONE; Toshiba Medical Systems, Tokyo, Japan) were used at the study hospitals, as previously reported^6,11^. Scans were obtained using 1.0 or 2.0 mm-thick sections of contiguous images from the apex to the lung base. Images were captured at a-600 HU (level) and 1600 HU (width) window setting. If a patient underwent CT before referral to our hospital, the CT features from the images taken at the referring institutes were evaluated.

The two respiratory medicine specialists blinded to clinical information independently evaluated the chest HRCT features, which included emphysematous lesions, GGOs, airspace consolidation, reticular shadows, centrilobular nodules, air bronchograms, cavities, bronchial wall thickening, and bronchiectasis. The investigators determined the distribution of GGOs and/or airspace consolidation in three areas in both lungs. A lingular segment was taken at the middle area of the left lung.

Furthermore, chest HRCT images reconstructed using the mediastinal settings were used to evaluate the presence of pleural effusion and hilar lymphadenopathy (with a short-axis diameter larger than 1 cm). Any disagreement in any findings was resolved by consensus.

### Statistical analyses

Statistical analyses were performed using the IBM SPSS statistics version 24 software package (IBM Japan, Tokyo, Japan). Unpaired Mann–Whitney *U*-test was used for continuous data, and χ^2^-test was used for categorical data to compare the two groups, except when the number of expected cells was less than five; in this case, Fisher’s exact test was used. Interobserver agreement was assessed by kappa value analysis. For two-tailed analyses, 95% confidence intervals were calculated. For all analyses, a *P* value < 0.05 was considered statistically significant.

Because the baseline characteristics of patients with pulmonary TB and those with non-TB CAP were different, propensity score matching was performed using a one-to-one matching protocol without replacement and a caliper width equal to 0.25 of the standard deviation of the logit of the propensity score. Standardized differences were estimated for all baseline covariates before and after matching.

### Ethical approval

The study protocol was approved by the institutional ethics committee of Oita University Hospital (approval number: 2045; approval date: March 22, 2021).

## Results

### Baseline characteristics of patients with pulmonary TB and those with non-TB CAP

Of 169 and 249 elderly patients with bacteriologically confirmed pulmonary TB or non-TB CAP, 159 (94%) and 238 (96%) underwent chest CT within two weeks before or after admission, respectively. After excluding patients who did not meet the CAP criteria, 151 patients with pulmonary TB and 238 patients with non-TB CAP were eventually included. Patients with pulmonary TB were significantly younger, and among them, more individuals had better performance status and complications of malignancy or diabetes. There were fewer patients with respiratory failure compared to those with non-TB CAP, as shown in Table 1. After the propensity score matching, the backgrounds of both groups became identical.

**Table 1:** Clinical characteristics of elderly patients with pulmonary tuberculosis and those with non-tuberculosis community-acquired pneumonia (CAP).

	Unmatched			Matched		
	Pulmonary TB (n = 151)	Non-TB CAP (n = 238)	p value	Pulmonary TB (n = 133)	Non-TB CAP (n = 133)	p value
Female	79 (52)	119 (50)	0.656	68 (51)	62 (47)	0.462
Age, years old	83 (79–89)	86 (80–91)	0.027	84 (79–89)	85 (77–90)	0.307
BMI, kg/m^2^	18.8 (16.7–20.5)	18.6 (16.7–21.8)	0.083	18.9 (16.8–20.7)	18.7 (16.9–22.6)	0.054
Performance status	3 (2–4)	4 (2–4)	< 0.001	3 (2–4)	3 (2–4)	0.782
Respiratory failure	53 (35)	109 (46)	0.012	49 (37)	43 (32)	0.439
Smoker	31 (28)	75 (32)	0.467	28 (29)	50 (38)	0.152
COPD	11 (7)	26 (11)	0.228	11 (8)	17 (13)	0.222
Heart failure	32 (21)	50 (21)	0.965	28 (19)	25 (21)	0.645
Cerebrovascular disease	34 (23)	63 (27)	0.380	31 (23)	30 (23)	0.884
Malignancy	27 (18)	10 (4)	< 0.001	10 (8)	10 (8)	1.000
Diabetes mellitus	29 (19)	28 (12)	0.043	26 (20)	19 (14)	0.252

Data are presented as number (%) or median (interquartile range).

*BMI* body mass index, *CAP* community-acquired pneumonia, *COPD* chronic obstructive pulmonary disease, *TB* tuberculosis.

Bacterial pathogens were isolated from 59 of the 133 matched patients with non-TB CAP (44%). The isolated pathogens were *Streptococcus pneumoniae* (n = 8), *Moraxella catarrhalis* (n = 8), *Klebsiella pneumoniae* (n = 6), *Haemophilus influenzae* (n = 6), methicillin-susceptible *Staphylococcus aureus* (n = 5), methicillin-resistant *Staphylococcus aureus* (n = 4), *Escherichia coli* (n = 4), *Pseudomonas aeruginosa* (n = 3), *Klebsiella oxytoca* (n = 3), *Prevotella* sp. (n = 3), *Streptococcus agalactiae* (n = 3), *Haemophilus parainfluenzae* (n = 1), and others (n = 5).

### Comparison of CXR features between patients with pulmonary TB and those with non-TB CAP

The kappa values of the chest radiograph findings were as follows: 0.828 for GGOs, 0.870 for airspace consolidation, 0.969 for nodules, and 0.854 for cavitary lesions.

As shown in Table 2, nodules and cavities were more frequently observed in patients with pulmonary TB. Significantly more lesions were also involved in patients with pulmonary TB. Infiltrates were more commonly distributed in the upper and middle lung fields of patients with pulmonary TB.

**Table 2:** Chest X-ray features of elderly patients with pulmonary tuberculosis and those with non-tuberculosis CAP.

	Pulmonary TB (n = 133)	Non-TB CAP (n = 133)	p value	NPV	PPV
GGO	96 (72)	99 (75)	0.603	0.471	0.492
Consolidation	97 (73)	87 (66)	0.215	0.556	0.527
Cavity	27 (20)	11 (8)	0.005	0.533	0.711
Nodule	35 (26)	16 (12)	0.003	0.542	0.686
**Distributions of GGO and/or consolidation**					
Number of segments	3 (2–5)	2 (1–3)	< 0.001		
Bilateral lungs	119 (90)	118 (89)	0.983	0.500	0.502
Right lung	107 (81)	101 (77)	0.436	0.544	0.514
Left lung	85 (64)	64 (49)	0.011	0.586	0.570
Upper field	101 (76)	62 (47)	< 0.001	0.686	0.620
Middle field	94 (71)	74 (56)	0.014	0.598	0.560
Lower field	91 (68)	97 (74)	0.364	0.455	0.484

Data are presented as number (%).

*CAP* community-acquired pneumonia, *TB* tuberculosis, *NPV* negative predictive value, *PPV* positive predictive value, *GGO* ground-glass opacities.

### Comparison of chest HRCT features between patients with pulmonary TB and those with non-TB CAP

The kappa values of the CT findings were as follows: 0.868 for emphysematous lesions, 0.903 for GGOs, 0.849 for airspace consolidation, 0.619 for reticular shadow, 0.924 for centrilobular nodules, 0.901 for air bronchograms, 0.933 for cavitary lesions, 0.763 for bronchial wall thickening, 0.786 for bronchiectasis, 0.970 for pleural effusion, and 0.651 for lymphadenopathy.

As shown in Table 3, centrilobular nodules, air bronchograms, cavities, pleural effusion, and lymphadenopathy were more frequently observed in patients with pulmonary TB. In contrast, GGOs and bronchial wall thickening were more frequently found in patients with non-TB CAP. Significantly fewer lobes were involved in patients with pulmonary TB. Infiltrates were less commonly distributed bilaterally in the middle and lower lobes in patients with pulmonary TB.

**Table 3:** High-resolution computed tomography (HRCT) features of elderly patients with pulmonary tuberculosis and those with non-tuberculosis CAP.

	Pulmonary TB (n = 133)	Non-TB CAP (n = 133)	p value	NPV	PPV
Emphysematous lesions	38 (29)	30 (23)	0.261	0.480	0.441
GGO	72 (54)	110 (83)	< 0.001	0.274	0.396
Airspace consolidation	109 (82)	105 (79)	0.536	0.538	0.509
Reticular shadow	8 (6)	16 (12)	0.087	0.483	0.333
Centrilobular nodule	93 (70)	37 (28)	< 0.001	0.706	0.715
Air bronchogram	80 (60)	21 (16)	< 0.001	0.679	0.792
Cavity	56 (42)	2 (2)	< 0.001	0.630	0.966
Bronchial wall thickening	31 (23)	58 (44)	< 0.001	0.424	0.348
Bronchiectasis	30 (23)	31 (23)	0.884	0.498	0.492
Pleural effusion	46 (35)	29 (22)	0.021	0.545	0.613
Enlargement of mediastinum and/or hilar lymph node	52 (39)	31 (23)	0.005	0.557	0.627
**Distributions of GGO and/or consolidation**					
Number of lobe involvements	2 (1–4)	3 (2–5)	0.002		
Bilateral lungs	64 (48)	96 (72)	< 0.001	0.349	0.400
Right lung	96 (72)	121 (91)	< 0.001	0.245	0.442
Left lung	83 (62)	113 (85)	< 0.001	0.286	0.423
Upper lobes	81 (61)	77 (58)	0.617	0.519	0.513
Middle lobes	80 (60)	93 (70)	0.095	0.430	0.462
Lower lobes	79 (59)	114 (86)	< 0.001	0.260	0.409

Data are presented as number (%).

*CAP* community-acquired pneumonia, *TB* tuberculosis, *NPV* negative predictive value, *PPV* positive predictive value, *GGO* ground-glass opacities.

Based on multivariate analysis, the presence of centrilobular nodules, air bronchograms, and cavities and the absence of GGOs and bronchial wall thickening were significantly associated with pulmonary TB (Table 4). However, the NPV of centrilobular nodules, air bronchograms, and cavities for pulmonary TB was not high, whereas the positive predictive value (PPV) of cavities was excellent.

**Table 4:** Multivariate analysis for the identification of pulmonary tuberculosis among elderly patients with CAP based on HRCT features.

	Odd ratio	95% CI	p value
GGO	0.260	0.119–0.570	< 0.001
Centrilobular nodule	6.049	2.930–12.491	< 0.001
Air bronchogram	5.817	2.733–12.379	< 0.001
Cavity	25.635	5.550–118.406	< 0.001
Bronchial wall thickening	0.236	0.103–0.544	< 0.001
Pleural effusion	2.175	0.995–4.752	0.051
Enlargement of mediastinum and/or hilar lymph node	1.637	0.766–3.493	0.203

*CI* confidence interval, *GGO* ground-glass opacities.

## Discussion

This study showed that the NPV and PPV of some HRCT features for pulmonary TB were greater than those of plain CXR features. Detailed HRCT findings may contribute to a precise diagnosis; centrilobular nodules, air bronchograms, and cavities and the absence of GGOs and bronchial wall thickening were significantly associated with the identification of pulmonary TB among elderly patients with CAP.

The more frequent observation of centrilobular nodules and cavities in patients with pulmonary TB is consistent with previous studies^7,19^. These features reflect granulomatous lesions with caseous necrosis and are highly specific predictors of mycobacterial infection^20^. Air bronchograms have not been fully evaluated for differentiating pulmonary TB from non-TB CAP. However, this feature is commonly found in approximately 60% of patients with caseous pneumonia resulting from TB infection^11,21^. Air bronchograms are airless lung tissues surrounding a normal open airway, which resulted from any alveolar diseases^22^. Pulmonary TB develops when airborne mycobacteria are inhaled and reach the pulmonary alveoli. Inflammation may spread in alveolar area through the pores of Kohn or canals of Lambert, which may develop air bronchograms. In contrast, among elderly patients with non-TB CAP, air bronchograms are rarely found on chest radiography^23,24^. Most cases of non-TB CAP in the elderly are characterized by aspiration pneumonia^25,26^, and this pathogenesis may cause airway obstruction due to the aspirated material; this does not lead to the development of air bronchograms. In patients with pneumonia who were confirmed to have a swallowing dysfunction based on videofluorographic evaluation, 68% had a bronchopneumonia pattern without air bronchograms^27^. The bronchopneumonia pattern is also likely to be accompanied by bronchial wall thickening, which was more frequently observed in patients with non-TB CAP in the current study.

GGOs on HRCT images were more commonly seen in patients with non-TB CAP, and the findings of plain CXR were not significantly different. GGOs reflect various pathological findings, including interstitial edema, alveolitis, or acute infection^28^. Pulmonary TB generally develops more slowly than non-TB CAP. This chronic pathogenesis may explain why GGO is less frequently seen in patients with pulmonary TB.

Infiltration due to pulmonary TB is typically distributed predominantly in the upper lung fields^20^. Although the upper lung field distribution was more commonly seen in patients with pulmonary TB using plain CXR, HRCT showed no significant difference in the upper lobe distribution of pulmonary TB and non-TB CAP. The upper lung fields on plain CXR include the upper lobes and superior segment (S6) of the lower lobes, which may explain why upper lung “field” and not “lobe” was more commonly seen in patients with pulmonary TB. Furthermore, the infiltration in non-TB CAP tended to be diffusely distributed on HRCT images, as shown in Table 3. This distribution is probably influenced by aspiration pneumonia in bedridden patients. Infiltrations were distributed across multiple lobes in patients with aspiration pneumonia^27,29,30^, and these were less frequently seen in the upper lobes of elderly patients with TB^6^. This fact may also support the reason for the absence of a significant difference in the upper lobe distribution of both groups.

This study revealed some useful factors that differentiate pulmonary TB from non-TB CAP. However, in clinical practice, misdiagnosing pulmonary TB as non-TB CAP may lead to delays in initiating appropriate treatments and increase the risk of spreading TB in communities and hospitals. To minimize this, findings with a high NPV are necessary. Unfortunately, in this study, no such feature was found even when using HRCT images, whereas cavities had a high PPV for the identification of pulmonary TB among elderly patients with CAP. Although these features may be useful in distinguishing pulmonary TB from non-TB CAP, an AFB smear test would be necessary for all patients presenting as CAP to avoid missing the diagnosis of pulmonary TB.

The strength of this study was the adjustment for patient backgrounds by propensity score matching to reduce selection bias, focusing on cases in which forming a differential diagnosis is challenging based solely on patient backgrounds. However, this study had several limitations. First, radiological features in any respiratory diseases may be affected by levels of burden and universal health coverage. For example, when medical accessibility is difficult in high TB burden areas, the disease status would be worse when diagnosed. Japan is a medium-burden TB country, and the accessibility of patients to medical service is good due to the universal health insurance. Second, elderly patients over 65 years old were included, but the median ages were high; it was 84 years in patients with pulmonary TB and 85 years in patients with non-TB CAP. Therefore, our results may only apply to super-aged populations. However, the aging population of Japan and other developed countries is increasing at an alarming rate. Third, performing HRCT on all elderly patients with CAP to distinguish pulmonary TB might be too costly, whereas we usually undergo HRCT for almost all patients with pulmonary TB and non-TB CAP. However, HRCT is increasingly performed currently in clinical practice, especially in middle- or high-income countries. Thus, the results of this study would be informative for patients in the area. Finally, some patients with pulmonary TB could be co-infected with non-TB pathogens. This may bias the radiological features, but it is also challenging to determine whether the bacteria isolated from sputum represent infection or colonization.

In conclusion, among elderly patients with CAP, although some HRCT features can differentiate pulmonary TB from non-TB CAP, there were no useful findings that could rule out pulmonary TB with certainty. Routine AFB tests would be ideal to prevent the misdiagnosis of pulmonary TB as non-TB CAP.

## References

[1] Mori T, Leung CC (2010). Tuberculosis in the global aging population. Infect. Dis. Clin. North Am..

[2] Komiya K, Ishii H, Kadota J (2015). Healthcare-associated pneumonia and aspiration pneumonia. Aging Dis..

[3] Kolappan C, Gopi PG, Subramani R, Narayanan PR (2007). Selected biological and behavioural risk factors associated with pulmonary tuberculosis. Int. J. Tuberc. Lung Dis..

[4] Fujishima N (2019). A pitfall of treatment with tosufloxacin for pneumonia that might be lung tuberculosis. Internal Med. (Tokyo, Japan).

[5] Pinto LM (2013). Development of a simple reliable radiographic scoring system to aid the diagnosis of pulmonary tuberculosis. PLoS ONE.

[6] Goto A (2019). Factors associated with atypical radiological findings of pulmonary tuberculosis. PLoS ONE.

[7] Yeh JJ (2014). A high-resolution computed tomography-based scoring system to differentiate the most infectious active pulmonary tuberculosis from community-acquired pneumonia in elderly and non-elderly patients. Eur. Radiol..

[8] Qi M, Li PJ, Wang Y, Liang ZA (2021). Clinical features of atypical tuberculosis mimicking bacterial pneumonia. Open Med. (Wars).

[9] Liam, C. K., Pang, Y. K. & Poosparajah, S. Pulmonary tuberculosis presenting as community-acquired pneumonia. *Respirology (Carlton, Vic.)* **11**, 786–792. 10.1111/j.1440-1843.2006.00947.x (2006). 10.1111/j.1440-1843.2006.00947.x 17052309

[10] Mandell LA (2007). Infectious Diseases Society of America/American Thoracic Society consensus guidelines on the management of community-acquired pneumonia in adults. Clin. Infect. Dis..

[11] Kan, T. *et al.* Comparison of chest computed tomography features between pulmonary tuberculosis patients with culture-positive and culture-negative sputum for non-mycobacteria: A retrospective observational study. *Medicine (Baltimore)* **100**, e26897. 10.1097/md.0000000000026897 (2021). 10.1097/MD.0000000000026897 PMC8341271 34397866

[12] Kan T (2019). Impact of additional antibiotics on in-hospital mortality in tuberculosis isolated general bacteria: A propensity score analysis. J. Infect. Chemother..

[13] Komiya K (2020). A high C-reactive protein level and poor performance status are associated with delayed sputum conversion in elderly patients with pulmonary tuberculosis in Japan. Clin. Respir. J..

[14] Honjo K (2020). The impact of performance status on tuberculosis-related death among elderly patients with lung tuberculosis: A competing risk regression analysis. J. Infect. Chemother..

[15] Yoshikawa H (2021). Quantitative assessment of erector spinae muscles and prognosis in elderly patients with pneumonia. Sci. Rep..

[16] Goto A, Komiya K, Hiramatsu K, Kadota JI (2021). The Efficacy of Penicillins with β-lactamase Inhibitor or Cefmetazole against Pneumonia in which ESBL-Producing Bacteria were Isolated from Sputum. Infect. Chemother..

[17] Tanaka A (2021). Quantitative assessment of the association between erector spinae muscle and in-hospital mortality in elderly patients with pulmonary tuberculosis. BMC Res. Notes.

[18] Oken MM (1982). Toxicity and response criteria of the Eastern Cooperative Oncology Group. Am. J. Clin. Oncol..

[19] Chan CH, Cohen M, Pang J (1992). A prospective study of community-acquired pneumonia in Hong Kong. Chest.

[20] Pinto LM (2013). Scoring systems using chest radiographic features for the diagnosis of pulmonary tuberculosis in adults: A systematic review. Eur. Respir. J..

[21] Qu, H., Zhang, W., Yang, J., Jia, S. & Wang, G. The value of the air bronchogram sign on CT image in the identification of different solitary pulmonary consolidation lesions. *Medicine (Baltimore)* **97**, e11985. 10.1097/md.0000000000011985 (2018). 10.1097/MD.0000000000011985 PMC6392802 30170400

[22] Fleischner FG (1948). The visible bronchial tree; A roentgen sign in pneumonic and other pulmonary consolidations. Radiology.

[23] Park S, Hong YK, Joo SH, Choe KO, Cho SH (1999). CT findings of pulmonary tuberculosis presenting as segmental consolidation. J. Comput. Assist. Tomogr..

[24] Nambu A, Ozawa K, Kobayashi N, Tago M (2014). Imaging of community-acquired pneumonia: Roles of imaging examinations, imaging diagnosis of specific pathogens and discrimination from noninfectious diseases. World J. Radiol..

[25] Teramoto S (2008). High incidence of aspiration pneumonia in community- and hospital-acquired pneumonia in hospitalized patients: A multicenter, prospective study in Japan. J. Am. Geriatr. Soc..

[26] Komiya K (2016). Prognostic implications of aspiration pneumonia in patients with community acquired pneumonia: A systematic review with meta-analysis. Sci. Rep..

[27] Komiya K (2013). Computed tomography findings of aspiration pneumonia in 53 patients. Geriatr. Gerontol. Int..

[28] Battista G, Sassi C, Zompatori M, Palmarini D, Canini R (2003). Ground-glass opacity: interpretation of high resolution CT findings. Radiol. Med..

[29] Komiya K (2013). Impact of aspiration pneumonia in patients with community-acquired pneumonia and healthcare-associated pneumonia: A multicenter retrospective cohort study. Respirology.

[30] Scheeren B, Gomes E, Alves G, Marchiori E, Hochhegger B (2017). Chest CT findings in patients with dysphagia and aspiration: A systematic review. J. Bras. Pneumol..

